# Multi-*e*GO: Model Improvements toward the Study of Complex Self-Assembly Processes

**DOI:** 10.1021/acs.jctc.3c01182

**Published:** 2023-12-28

**Authors:** Fran Bačić Toplek, Emanuele Scalone, Bruno Stegani, Cristina Paissoni, Riccardo Capelli, Carlo Camilloni

**Affiliations:** † Dipartimento di Bioscienze, Università degli Studi di Milano, Via Celoria 26, 20133 Milano, Italy; ‡ Department of Chemistry, Dartmouth College, Hanover, New Hampshire 03755, United States

## Abstract

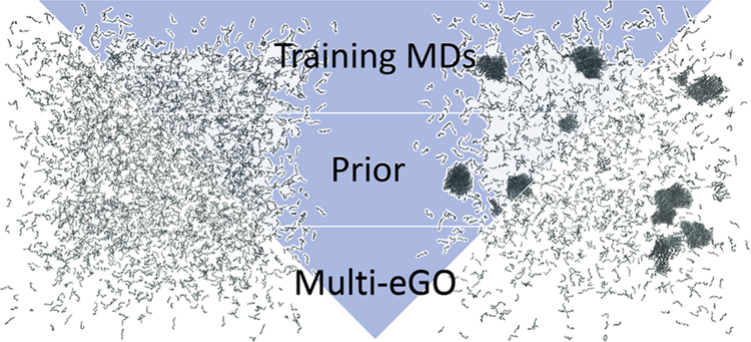

Structure-based models have been instrumental in simulating protein folding and suggesting hypotheses about the mechanisms involved. Nowadays, at least for fast-folding proteins, folding can be simulated in explicit solvent using classical molecular dynamics. However, other self-assembly processes, such as protein aggregation, are still far from being accessible. Recently, we proposed that a hybrid multistate structure-based model, multi-*e*GO, could help to bridge the gap toward the simulation of out-of-equilibrium, concentration-dependent self-assembly processes. Here, we further improve the model and show how multi-*e*GO can effectively and accurately learn the conformational ensemble of the amyloid β42 intrinsically disordered peptide, reproduce the well-established folding mechanism of the B1 immunoglobulin-binding domain of streptococcal protein G, and reproduce the aggregation as a function of the concentration of the transthyretin 105–115 amyloidogenic peptide. We envision that by learning from the dynamics of a few minima, multi-*e*GO can become a platform for simulating processes inaccessible to other simulation techniques.

## 1 Introduction

Molecular dynamics (MD) simulations, based on conventional transferable molecular mechanics force fields, have become a standard tool in biological research thanks to their ability to resolve the atomic details of many molecular processes.^1^ This success is the result of a combination of increased computational resources and associated software, improved sampling methods, more accurate force fields, and better methods for integrating simulations with experimental information.^2^ In addition, intrinsically disordered proteins (IDPs) or regions have highlighted the need to complement the structure with dynamics by challenging the sequence-structure paradigm.^3^ Notably, the revolution in AI-based structure prediction tools has further widened the scope of simulations by allowing the study of systems whose structure has not yet been experimentally determined.^4,5^

Despite these advances, conventional atomistic MD simulations are still limited to the study of relatively few molecules on a time scale of tens of microseconds.^6−8^ To overcome size and time scale limitations, one can limit the scope of the simulation technique to specific subdomains and use simplified models.^9^ This strategy is exemplified by the Martini force field,^10^ which focuses mainly on folded proteins and lipid membranes and allows the simulation of large protein complexes in a realistic environment. More recently, other simplified models have emerged, focusing on IDPs and their interaction processes in the context of liquid–liquid phase separation.^11−14^ We are also seeing the first examples of simplified models resulting from machine-learned potentials trained on classical force fields.^15^ Since the 1990s, structure-based models have played a key role in elucidating the protein folding process by learning a system-dependent potential that should have an absolute energy minimum centered on the chosen folded structure.^16^

Recently, building on the observation that the amyloid structure could be the most stable one that protein molecules can adopt under physiological conditions,^17^ we have revisited structure-based models to incorporate information from multiple minima with the aim of describing the aggregation of a peptide into an amyloid fibril.^18^ Our model, called multi-*e*GO, allowed us to qualitatively capture the experimental macroscopic features of protein aggregation, including kinetics and fibril morphology, and to shed light on the microscopic features of the process.^18^

Multi-*e*GO is a hybrid transferable/structure-based model defined from a combination of simulations and structures. Only the heavy atoms (nonhydrogens) are included to maintain atomic resolution. Bonds, angles, dihedrals, and default *C*^(12)^ values are based on the GROMOS54a7 force field,^19^ being already optimized without nonpolar hydrogens, dihedral terms for the φ and ψ torsions and some 1–4 nonbonded interactions (i.e., pairs of atoms that are separated by three consecutive covalent bonds) are specifically reoptimized. Attractive nonbonded interactions are obtained from either a PDB structure or an MD simulation and parametrized using the Lennard–Jones (LJ) potential. The structure-based potential is defined by pairs of atoms within a 0.55 nm cutoff, with an interaction strength rescaled using contact probabilities. The ε_i,j_ of the LJ potential was heuristically defined as
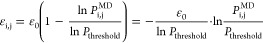
1where ε_0_ is the maximum interaction energy provided in the input, *P*_i,j_^MD^ is the population for the contact between atoms i,j as obtained from a training MD simulation, and *P*_threshold_^MD^ is a minimum population that should be considered. Such LJ parametrization allowed an increase in protein flexibility when combined with the transferable bonded terms.

**Figure 1:**
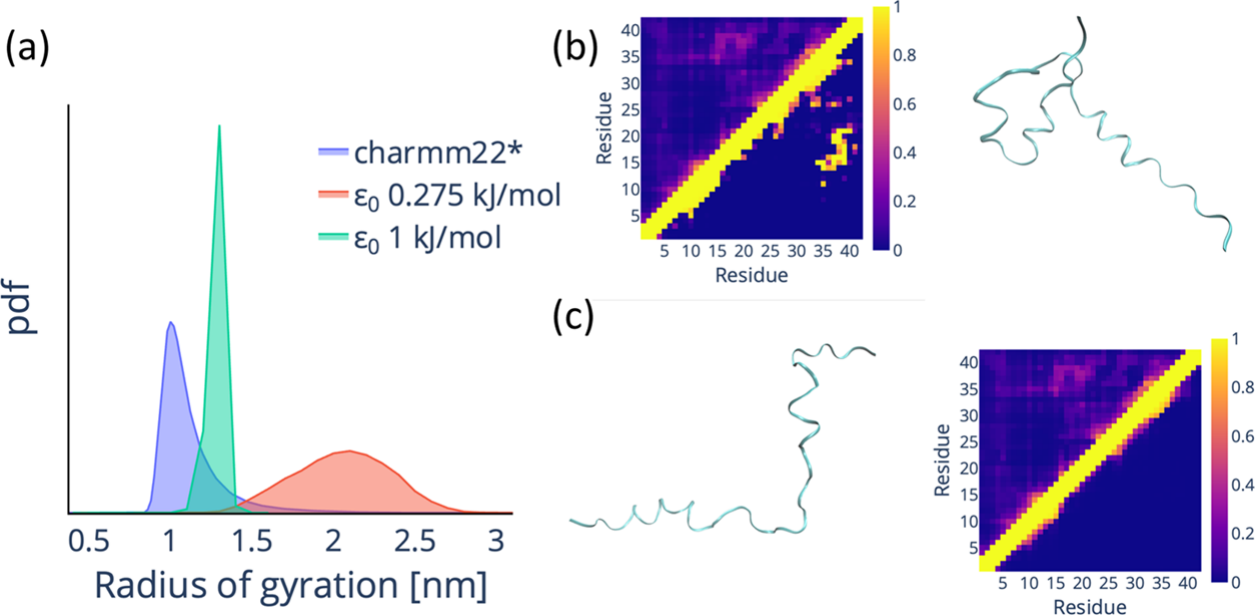
Original multi-*e*GO fails to reproduce the conformational dynamics of the Aβ42 monomer. (a) Probability density function (pdf) for the backbone radius of gyration of Aβ42 for a training simulation (charmm22*, blue)^22^ and for an original multi-*e*GO simulation with ε_0_ = 0.275 kJ/mol (red) and ε_0_ = 1 kJ/mol (green). (b) Comparison of the per-residue probability contact map for the training and original multi-*e*GO simulation with ε_0_ = 1 kJ/mol and a representative structure from the multi-*e*GO simulation. The colored bar represents the contact probability. (c) Comparison of the per-residue probability contact map for the training and original multi-*e*GO simulation with ε_0_ = 0.275 kJ/mol and a representative structure from the multi-*e*GO simulation. The color bar represents the contact probability.

Encouraged by our previous results, we built a multi-*e*GO model to simulate the amyloid β42 peptide (Aβ42)^20,21^ using previously published MD trajectories^22^ for training (with the only difference that we set *P*_threshold_ to 0.01 compared to the 0.09 value used in our previous work). As shown in Figure 1, the original multi-*e*GO parametrization does not allow us to capture the training Aβ42 conformational ensemble with any ε_0_ value. Our interpretation is that this is due to the imbalance between the highly populated local contacts and the very weakly populated contacts between distant residues, which are typical IDPs such as Aβ42. Our hypothesis was supported by the impossibility of learning contacts using the smaller epsilon value and the impossibility of getting to a compact structure at a larger epsilon. These results indicate the need to improve eq 1 to better account for the polymer properties.

In what follows, we therefore present a reformulation of the multi-*e*GO model in which following Bayesian statistics, we update a prior polymer model with additional information learned from training simulations. We show how this leads to a more complete description of attractive and repulsive interactions. We demonstrate that the updated multi-*e*GO can correctly learn the dynamics of an IDP such as Aβ42, can be used to describe the folding mechanism of a small protein such as the B1 immunoglobulin-binding domain of streptococcal protein G (GB1),^23^ and can still reproduce the recently published results on the aggregation of the transthyretin 105–115 amyloidogenic peptide (TTR_105–115_).^18,24^ We therefore propose this improved multi-*e*GO as a model that, using only the information that can be generated with conventional MD, can approximate processes on size and time scales that are orders of magnitude larger than the state of the art.

## 2 Theory

### 2.1 Multi-*e*GO: A Bayesian Reformulation

Equation 1 introduced above suggests, in the form on the right side, that the MD contact probabilities are weighted by a uniform, uninformative, prior distribution. Proteins are polymers with a local geometry described by the Ramachandran plot, and as such, the contact probabilities between atoms along the chain are influenced by the chain geometry. A more informative prior would be that of a self-avoiding chain with local geometries as close as possible to those of proteins. We call random coil (RC) probability distribution the contact pair distribution resulting from a simulation of such a model (cf., next section). Consideration of this new prior leads to a reformulated equation for the interaction energy
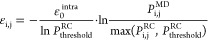
2where *P*_i,j_^MD^ and *P*_i,j_^RC^ are the fraction of frames with a native contact in the MD and RC simulations, respectively. In this new parametrization, the information on the prior model is retained until a minimum value *P*_threshold_^RC^ is reached. This equation is the core of the new multi-*e*GO, and it is important to note that it is meant to account in general for any prior assumptions, which means that other prior models can be used for specific problems, as will be shown later for intermolecular interactions. Another important consequence of this formula is that if *P*_i,j_^RC^ > *P*_i,j_^MD^, the sign of the energy changes, indicating the need to introduce repulsive interactions. Notably, this is similar to approaches previously introduced to reweight statistical potentials used in protein structure prediction.^25^

### 2.2 Multi-*e*GO Prior, Random Coil, Model

In the new formulation of multi-*e*GO, the prior is essential to define the nonbonded interaction strength. Therefore, we reoptimized the local geometries of the model to obtain the most informative prior possible by reparametrizing the default *C*^(12)^ parameters of the LJ potential, the 1–4 excluded volume pairs, and the dihedral parameters. First, bonds, angles, and proper and improper dihedrals were taken from the GROMOS54a7^19^ force field as before. *C*^(12)^ values for the GROMOS54a7 atom types were scaled down so that the atomic radius is defined as the distance at which the repulsion is equal to the thermal energy at 300 K (*k*_B_*T* = 2.49 kJ/mol). In the case of oxygen–oxygen interactions, we introduced a scaled-up *C*^(12)^ (11.4-fold larger than the obtained by the procedure described above) to account for their strong electrostatic repulsion. We then introduced new 1–4 pairs, defined only by their *C*^(12)^, to account for the correct local excluded volume potential. The newly introduced pairs include C_–1_–C_β_, C_β_–O, C_β_–N_+1_, N–N_+1_, C–C_+1_, C–C_γ_, and N–C_γ_, most of which were added based on.^26,27^ 1–4 *C*^(12)^ were fine-tuned using different dipeptides to match a corresponding target Ramachandran distribution, as obtained from an explicit solvent simulation using the CHARMM22* force field.^28^ Finally, the parameters corresponding to the φ and ψ backbone dihedrals were optimized to minimize the difference between the Ramachandran distributions. The dipeptides used were glycine dipeptide, proline dipeptide, alanine dipeptide, and valine dipeptide, the latter two being used to represent residues with small and bulky side chains, respectively. The small amino acids include alanine, serine, and threonine, while the valine dipeptide was chosen as a proxy for the other 15 amino acids. This distinction was necessary because bulky amino acids behave differently toward the upper left corner (extended β conformation) of the Ramachandran distribution. Bulky amino acids generally have less extended β conformations and a smoother β distribution overall, requiring separately optimized dihedral parameters.

### 2.3 Attractive Interactions

Nonbonded interactions in multi-*e*GO are implemented using the LJ potential, i.e., , with repulsive interactions defined only by the value *C*^(12)^ = 4εσ.^12^ To generate the model potential, we then set both ε and σ. As already introduced above, ε is defined as in eq 2 from the ratio between the contact probability of a pair of atoms as observed in the training MD simulation and in the corresponding RC simulation (i.e., a simulation based only on the multi-*e*GO prior model). As a rule, the RC simulation must be performed at the same temperature as the corresponding training MD. The multi-*e*GO simulation can then be performed at a different temperature, but given the simplified nature of the interactions, the extrapolation in temperature will add additional approximations. The contact probability between two atoms is defined here as *P*_i,j_ = ∫_0_^*R*_i,j_^cut^^*P*_i,j_(*x*)d*x*, i.e., the probability of observing the i,j pair in the distance range [0, *R*_i,j_^cut^]. In the original multi-*e*GO, *R*_i,j_^cut^ was set to 0.55 nm irrespective of the i,j pair, but it should be noted that in a conventional MD simulation, certain atom types can hardly form interactions in this distance due to their size, e.g., α carbons, while for other pairs, this distance is far too permissive, e.g., nitrogen–oxygen forming a hydrogen bond. Therefore, we have introduced a variable , where the cutoff factor *f*_cut_ = 1.45 is chosen so that the atom forming pairs are subject to 80% of the standard LJ attraction. In LJ, σ is related to the position of the minimum of the potential as , where *r*_min_ should be considered as the interaction length. To estimate *r*_min_ from our simulations, we use an exponential averaging with a resolution of 0.1 nm
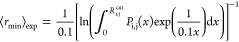
3

From eq 2, it is clear that ε can tend to zero if *P*_i,j_^RC^–*P*_i,j_^MD^, but as the value of ε decreases, the LJ potential becomes increasingly permissive with respect to distances shorter than σ, thus suggesting that ε should not become too small to prevent atoms from exploring unphysical conformations. Consequently, we can introduce a minimum fraction of ε_0_^intra^ below, which we do not define as an attractive interaction. Given this minimum fraction *f*_ε_ and eq 2, an attractive interaction is defined if and only if *P*_i,j_^MD^ > (*P*_threshold_^RC^)^−*f*_ε_^ max(*P*_i,j_^RC^, *P*_threshold_^RC^). Here, we set *f*_ε_ = 0.2 by default so that ε is not less than 0.2ε_0_^intra^. However, in this way, attractive interactions can still be defined for pairs of atoms with very small *P*_i,j_^MD^, for which the estimate of both *P*_i,j_^MD^ and *r*_min_ may be poor due to limited statistical sampling. We would therefore like to add an additional condition for learning attractive interactions, namely, *P*_i,j_^MD^ > *P*_threshold_^MD^. Given a value for *P*_threshold_^MD^, it is possible to define

 in such a way that the two conditions do not overlap. Now let us consider the case of two different training MD simulations, one for a system exploring a very homogeneous conformational ensemble and the other exploring a very heterogeneous one. Using a single *P*_threshold_^MD^ can lead to learning irrelevant contacts in the first case and discarding relevant ones in the second. To obtain an adaptive *P*_threshold_^MD^, we instead set *P*_learn_ as the fraction of the total contact population to learn from a training simulation. We sort all *P*_i,j_^MD^ in descending order and normalize them by the total contact population ∑*P*_i,j_^MD^, and then we take *P*_threshold_^MD^ as the *P*_i,j_^MD^ value associated with a cumulative sum equal to *P*_learn_. Our default choice is *P*_learn_ = 0.9995, where a value too small would result in poor learning of the training simulations, while a value too large may result in learning of numerical noise.

### 2.4 Repulsive Interactions

Equation 2 can also lead to negative ε, as mentioned above, but LJ is not well-defined in this case. Instead, repulsive interactions can be implemented by setting *C*^(6)^ = 0 and *C*^(12)^ > 0. We derived a general formula to update our default *C*^(12)^ (cf. Section 2.2) by observing the following approximate relationship between the probability of a contact and its interaction length *r*_min_ in the RC and MD simulations
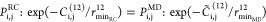
4where *C̃*_i,j_^(12)^ is the updated effective value we wish to set to reproduce the training MD simulation. From the above relationship, we can see that
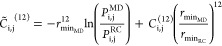
5which we regularize as in eq 2 to obtain
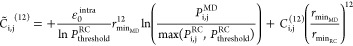
6

Equation 6 is applied whenever *P*_i,j_^MD^ < max (*P*_i,j_^RC^, *P*_threshold_^RC^) but greater than 0. Here, the second term of the sum allows the standard *C*_i,j_^(12)^ to be scaled up or down, while the first term is added on top. We also consider the case of (*P*_threshold_^RC^)^−*f*_ϵ_^ max (*P*_i,j_^RC^, *P*_threshold_^RC^) > *P*_i,j_^MD^ > max (*P*_i,j_^RC^, *P*_threshold_^RC^); in this case, we use the relationship because the first term would have a negative sign and could result in meaningless *C*_i,j_^(12)^ coefficients. Importantly, with respect to the case of attractive interactions, it is now possible for *P*_i,j_^MD^ and/or *P*_i,j_^RC^ to be less than *P*_threshold_^MD^, in which case the corresponding *r*_min_ is set to *R*_i,j_^cut^. Finally, to avoid unphysical interactions, the learned *C*_i,j_^(12)^ is limited to between 1/10 and 20 times the default one. 1–4 interactions are rescaled according to the same rules, but to avoid possible distortions of the Ramachandran space, their *C*_i,j_^(12)^ values cannot change more than a factor of 1.5. For all of the pairs of atoms not included in the attractive or repulsive cases, the prior *C*_i,j_^(12)^ is retained.

### 2.5 Intermolecular Interactions

Intramolecular geometries are generally compatible with intermolecular geometries, while the opposite is not true (i.e., some intermolecular contacts cannot be formed intramolecularly because of the constraints imposed by the polymer geometry). Therefore, in multi-*e*GO, when we learn an intramolecular interaction, it is applied intermolecularly, while the opposite is not true. If both intermolecular and intramolecular interactions are learned for the same pair of atoms, then both are retained and applied under the appropriate conditions. To estimate the intermolecular contact probabilities and interaction lengths, we analyze a training simulation containing *N* copies of a molecule for the probability that a pair of atoms form at least one intermolecular contact per molecule in each frame. This follows from the assumption that the contact strength should not depend on the coordination, i.e., the interactions are simply two-body. The interaction length for a pair is calculated using eq 3 over the distribution of the intermolecular pair distances. The interaction strength is then set according to eqs 2 and 6 with the possibility of setting ε_0_^inter^ ≠ ε_0_^intra^, but using an ad hoc prior model. The intermolecular prior model should estimate the probability of trivial intermolecular interactions resulting only from random collisions associated with the shape of the molecules and their concentration. In general, to avoid any issue in the entropic contributions of the model, our current approach is to run a simulation at the same concentration as the one we want to run in production, using a force field trained only for intramolecular interactions. It should be noted that an intermolecular prior model is only needed to learn intermolecular interactions specifically, not for the intramolecular model applied intermolecularly.

### 2.6 Learning from Multiple MD Simulations

A key feature of multi-*e*GO is its ability to learn from multiple MD simulations, generally associated with different free energy minima of the system. A prototypical example is that of amyloid fibrils, where we can provide for training a simulation of the monomer protein in solution, the free energy minimum at low concentration, and that of the protein in an amyloid fibril, the free energy minimum at high concentration. When merging contacts learned from different sources, we follow the following rules: (1) among multiple contacts for a given i,j pair, we chose the one with the shortest estimated interaction length as defined above (that is, for pairs with *P*_i,j_ > *P*_threshold_^MD^, we use eq 3, otherwise *r*_min_ = *R*_i,j_^cut^); (2) among several attractive and repulsive contacts for a given i,j pair with the same *r*_min_, we chose the attractive one with the largest ε; and (3) among several repulsive contacts for a given i,j pair, we chose the one with the smallest *C*_i,j_^(12)^. Another implemented option is to set an ensemble as the check data set. Setting an ensemble as such forces multi-*e*GO to perform a check on repulsive interactions to ensure compatibility with the check data set. The *C*_i,j_^(12)^ of repulsive contacts for which *r*_min_^check^ < *r*_min_^train^ are rescaled by (*r*_min_^check^/*r*_min_^train^)^12^.

## 3 Simulations Details

All MD simulations were performed using the GROMACS^29^ software suite. Metadynamics^30^ simulations were performed using the PLUMED2 library.^31,32^ Unless explicitly stated, all simulations were performed using the same 4-step protocol consisting of (1) energy minimization using the steepest descent algorithm until the maximum force converges to a value <1000 kJ mol^–1^nm^–1^, (2) conjugate-gradient minimization until the maximum force converges to a value <10 kJ mol^–1^ nm^–1^, (3) positionally restrained relaxation for 4 ns at constant pressure and temperature, and (4) the production simulation. Explicit solvent MD simulations were performed using the leapfrog algorithm with a time step of 2 fs and LINCS restraints^33^ for hydrogen atoms. Nonbonded interactions are cut off at 1 nm using PME for long-range electrostatics.^34^ Temperature and pressure are controlled by stochastic velocity rescaling^35^ and cell rescaling^36^ algorithms, respectively. Multi-*e*GO simulations were performed using stochastic dynamics integration with a time step of 5 fs and a relaxation time of 25 ps. The cutoff for the LJ interactions is set specifically for each system as 2.5σ_max_. A 10% larger radius is used for the neighbor lists, which are updated every 20 steps.

All scripts and parameters to generate a multi-*e*GO force field are publicly available on GitHub (cf. Notes). All simulations performed in this work are publicly available via Zenodo (cf. Notes).

### 3.1 Aβ42

The training trajectories for Aβ42 are publicly available and published in ref. (22) They include 315 μs of sampling at 278 K. An RC simulation was performed at the same temperature for 1 μs. Different ε_0_ values were then tested to maximize the agreement with the target radius of the gyration probability distribution until an optimal value of 0.335 kJ/mol was found. Production multi-*e*GO simulations were run in triplicate at the same temperature for 2 μs each. Clustering analyses were performed with the cluster module of GROMACS using the gromos algorithm described in ref (37) using the root-mean-square deviation (RMSD) of the backbone atom positions as a metric and a cutoff of 0.8 nm.

### 3.2 Protein GB1

The explicit solvent training simulation was performed using the CHARMM22* force field^28^ in conjunction with the TIP3P water model.^38^ A dodecahedral box was constructed 0.7 nm from the protein surface to minimize the number of explicit water molecules. The system charge was neutralized using a NaCl concentration of 0.2 mM. After energy minimization and temperature and density equilibration, production was performed for 1 μs of simulations in the NPT ensemble (*T* = 300 K, *P* = 1 bar). The RC simulation was also performed for 1 μs at 300 K. To calibrate the multi-*e*GO energy scale ε_0_, we used the melting temperature and its microscopic implications. First, we performed metadynamics simulations at the experimental melting temperature^23^*T*_m_ = 360 K to determine the ε_0_ value at which the folded and unfolded states are equally populated. For these simulations, we used the all-atom RMSD with respect to the crystal structure as a collective variable, adding Gaussians every 500 steps, with an initial height of 1.2 kJ/mol, a bias factor of 15, and a width of 0.025 nm. Following the identification of an appropriate ε_0_ = 0.235 kJ/mol, we prepared 200 starting configurations extracted from the RC simulation. We then ran 200 independent simulations at 300 K until the folded structure (i.e., all-atom RMSD with respect to the crystal structure of less than 0.3 nm) was reached. The data were analyzed using Biotite.^39^

### 3.3 TTR_105–115_ Peptide

The training trajectory for TTR_105–115_ was performed in our previous work^18^ using the a99SB-disp force field,^40^ for 1.6 μs at 300 K. The RC simulation was performed for 500 ns at the same temperature. The ε_0_ for the intramolecular interactions of the multi-*e*GO simulation was tuned by maximizing the agreement of the radius of gyration probability distribution, and the best result was found for ε_0_ = 0.275 kJ/mol.

A training trajectory for the TTR_105–115_ fibril was performed using the 2M5M PDB structure,^41^ consisting of 84 monomers, in a box containing 23,000 water molecules. The system was parametrized using the CHARMM22* force field^28^ and the TIP3P water model.^38^ The fibril was found to be unstable, so the simulation was run with a position restraint on all of the backbone and Cβ carbons of the system for 150 ns. To weight the intermolecular interactions, 2 μs multi-*e*GO simulations of 80 monomers at concentrations of 13, 10, and 7 mM were performed, trained only on the monomer MD, with ε_0_ = 0.275 kJ/mol. The ε_0_ for the intermolecular interactions was also set at 0.275 kJ/mol after a stable fibril structure was verified. Aggregation kinetics simulations were set up to generate boxes of 4000 monomers at concentrations of 13, 10, and 7 mM. Three initial configurations were generated for each concentration and first equilibrated using the monomer-only force field. Simulations were then run at 310 K and followed until aggregation.

## 4 Results

### 4.1 Multi-*e*GO Can Reproduce the Conformational Ensemble of an Intrinsically Disordered Protein

**Figure 2:**
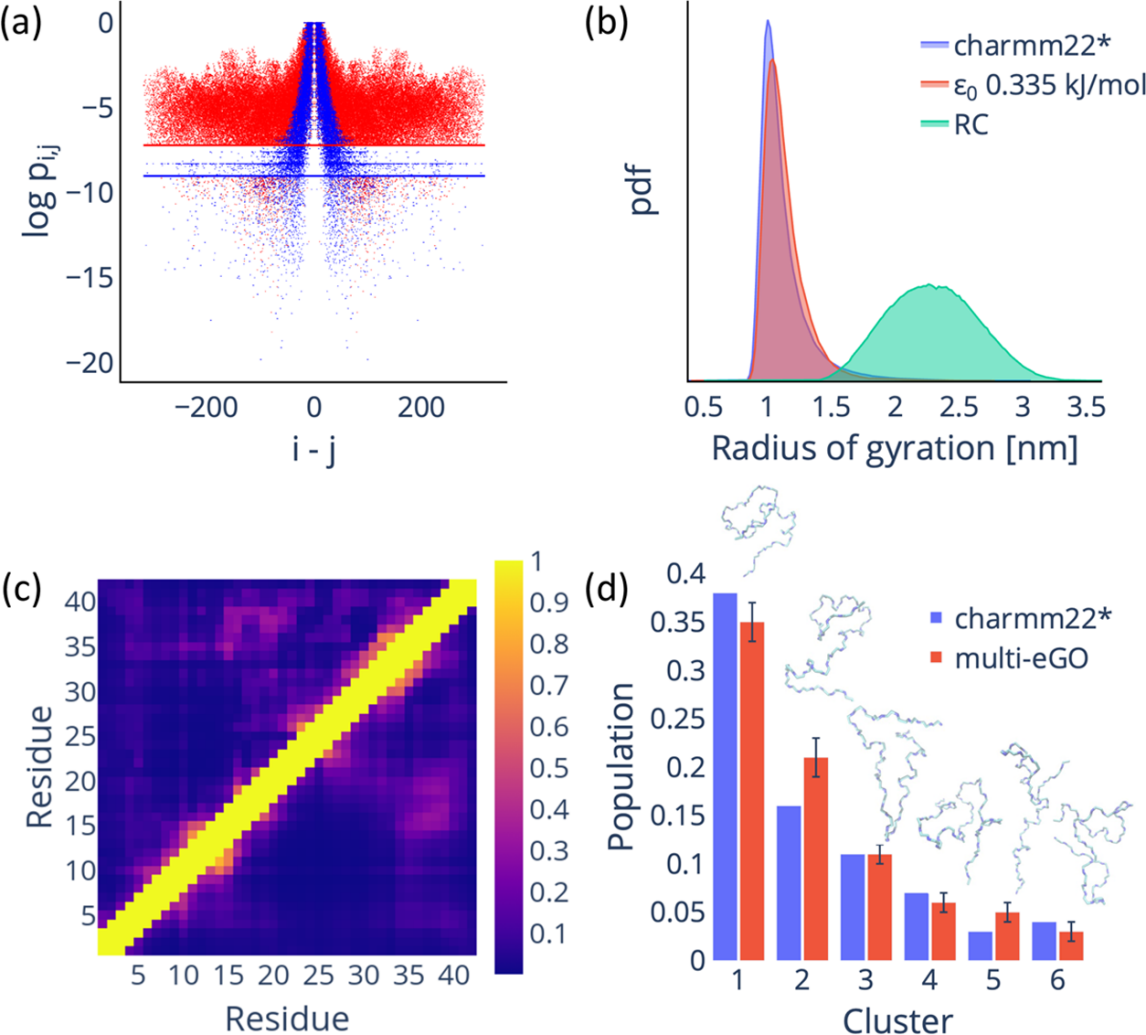
Reformulated multi-*e*GO model can reproduce the conformational dynamics of Aβ42 IDP. (a) Comparison of the atom-pair contact probabilities for the training (red dots) and RC (blue dots) simulations. The lines represent the *P*_threshold_^MD^ (red) and *P*_threshold_^RC^ (blue) values. (b) Comparison of the radius of gyration probability density function (calculated using the backbone atoms) for the training (blue), multi-*e*GO (orange), and RC (green) simulations. (c) The contact probability map per residue for the training (upper diagonal) and multi-*e*GO (lower diagonal) simulations. The color bar represents the contact probability. (d) Clustering analysis of multi-*e*GO and training simulations. The clusters are comparable in population size and in terms of order. A representative configuration is shown for each cluster.

In Figure 1, we have shown how our first multi-*e*GO implementation was unable to learn the heterogeneous conformational ensemble of Aβ42, as represented by its radius of gyration probability distribution and per-residue average contact map. We attribute this limitation to the imbalance between local and long-range interactions. In fact, the contact probability weighting equation heuristically introduced in our previous publication, eq 1 in the Introduction, can be rewritten as the ratio between a training probability and an uninformative uniform prior. By introducing a polymer-informed prior, cf. Section 2, we can weight each contact by its probability of forming as a consequence of the polymer geometry alone. As shown in Figure 2a, the distribution of the contact probabilities in the training simulation peaks for atoms close in sequence, decreasing with distance, but with some regions still showing low but non-negligible values with respect to *P*_threshold_^MD^ (e.g., 0.0007). On the contrary, the contact probabilities in the RC simulation are equally peaked for atoms close in sequence but systematically drop to zero for atoms further apart, at some point becoming smaller than the *P*_threshold_^RC^ value (e.g., 0.0001). The comparison of the two distributions allows a better understanding of how the multi-*e*GO model works by immediately highlighting that some distant contacts are very important compared to the probability of their formation by chance.

In Figure 2b, we show how this multi-*e*GO reformulation allows us to improve the agreement of the radius of gyration distribution not only to overlap at the maximum, where we find most of the closed conformations, but also to show how the simulations now match the tail, i.e., the open conformations with fewer contacts. The contact map analysis shown in Figure 2c further confirms the accuracy of the model, showing a strong resemblance to the training, with an average error of around 3.5%. Finally, we performed a clustering analysis^37^ on the combined trajectories of the multi-*e*GO simulation and the training to understand how the individual states are distributed. Remarkably, our analysis revealed that the six most representative clusters are equally represented in the training and multi-*e*GO conformational ensembles, as shown in Figure 2d.

Taking all of the results together, it is safe to say that the conformational ensemble sampled by multi-*e*GO is in quantitative agreement with the training ensemble used.

### 4.2 Multi-*e*GO Can Simulate the Folding Mechanism of a Small Protein

**Figure 3:**
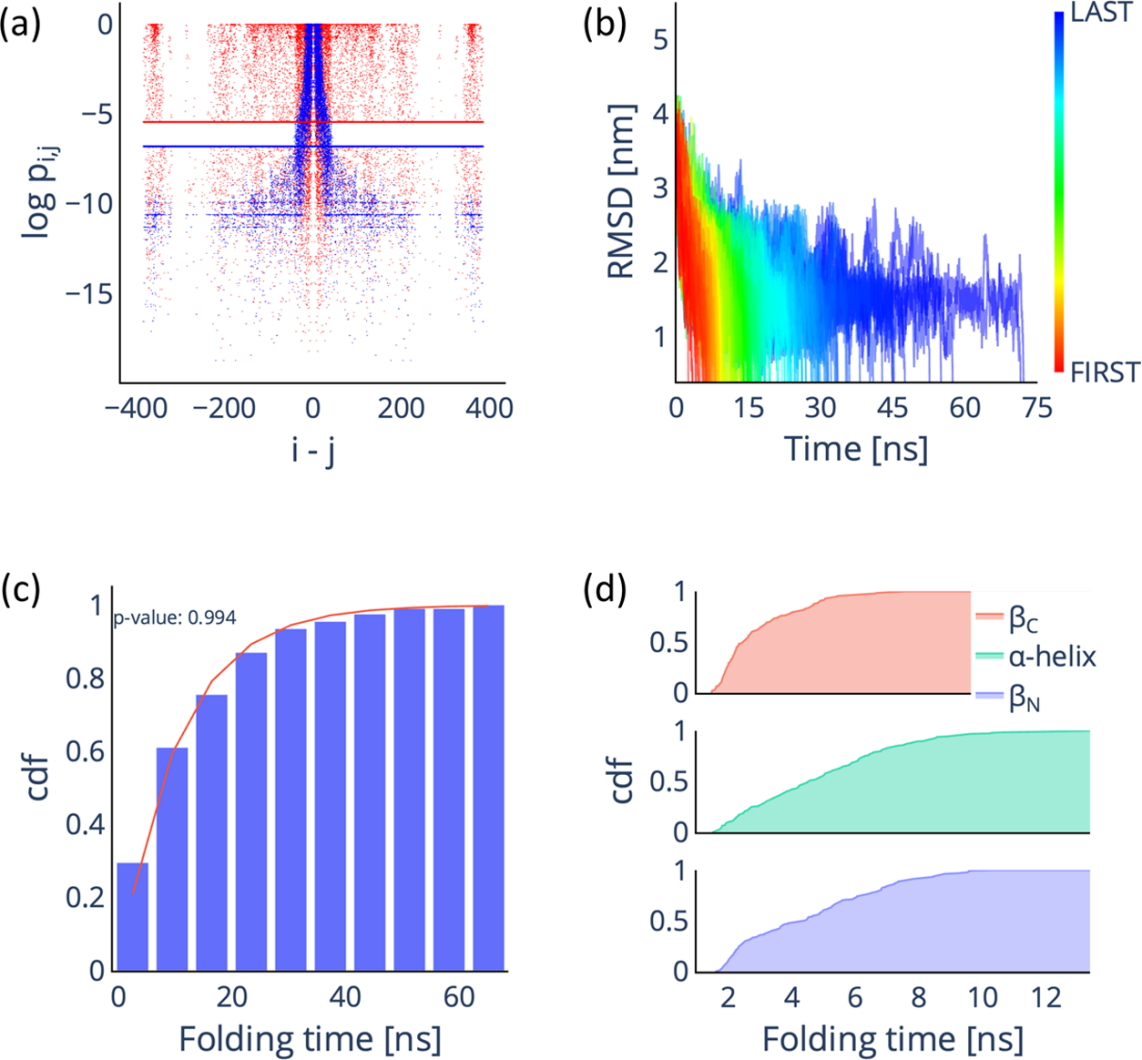
Multi-*e*GO can reproduce the folding mechanism of GB1. (a) Comparison of the atom-pair contact probabilities for the training (red dots) and RC (blue dots) simulations. The lines represent the *P*_threshold_^MD^ (red) and *P*_threshold_^RC^ (blue) values. (b) Time evolution of the RMSD with respect to the folded state for the 200 multi-*e*GO folding trajectories. (c) Cumulative distribution function (cdf) of the folding tim*e* distribution for the 200 multi-*e*GO folding trajectories (blue bars), fitted with the cdf of the Poisson distribution (red line) and associated *p*-value. (d) Cumulative distribution function for the folding times of the three GB1 secondary structure elements.

Having shown that multi-*e*GO can learn the conformational ensemble of an IDP, one can ask how it performs in a conventional structure-based modeling task, i.e., describing the folding mechanism of a folded protein. A system often studied by Go̅ models is protein GB1.^42−44^ The folding of this protein has been well characterized experimentally, showing that its C-terminal hairpin folds first, followed by its N-terminal one and the α-helix.^45^ To set up the model, we ran a training simulation of the folded protein and an RC simulation. Comparing the probability distribution of contact pairs in Figure 3a, we observe high probability contacts both close and farther apart in the sequence for the training simulation, as expected for a stable folded protein, while the RC simulation shows an identical behavior as previously shown for Aβ42, with highly probability contacts found only close in the sequence. After training a multi-*e*GO model to recover the experimental melting temperature of 360 K and setting ε_0_ to 0.235 kJ/mol, we ran 200 independent folding simulations, starting from RC configurations, see Figure 3b. The cumulative distribution function of the folding times in Figure 3c shows a typical Poisson distribution (*p*-value of 0.994 from a Kolmogorov–Smirnov test) with an average folding time of 17.4 ns. This time is nominal and when compared with the experimental time scale of 10 ms^45^ gives an idea of the speed-up achieved by multi-*e*GO.

To analyze the GB1 folding mechanism, we evaluated the folding time for each secondary structure element, i.e., the N- and C-terminal β-hairpins and the central α-helix, as the time at which each secondary structure was stably formed in the folding simulation; see Figure 3d. The analysis clearly highlights the C-terminal hairpin as the element that generally folds first. This is a way to measure the progress of the folding process and clearly indicates an asymmetric folding that starts preferentially from the C-terminal and ends with either the α-helix or the N-terminal hairpin, as previously reported.^43,44,46^ This result suggests that multi-*e*GO can correctly learn the native state energy of a folded protein and extrapolate about the folding mechanism.

### 4.3 Multi-*e*GO Can Still Qualitatively Describe the Aggregation Kinetics of TTR_105–115_

**Figure 4:**
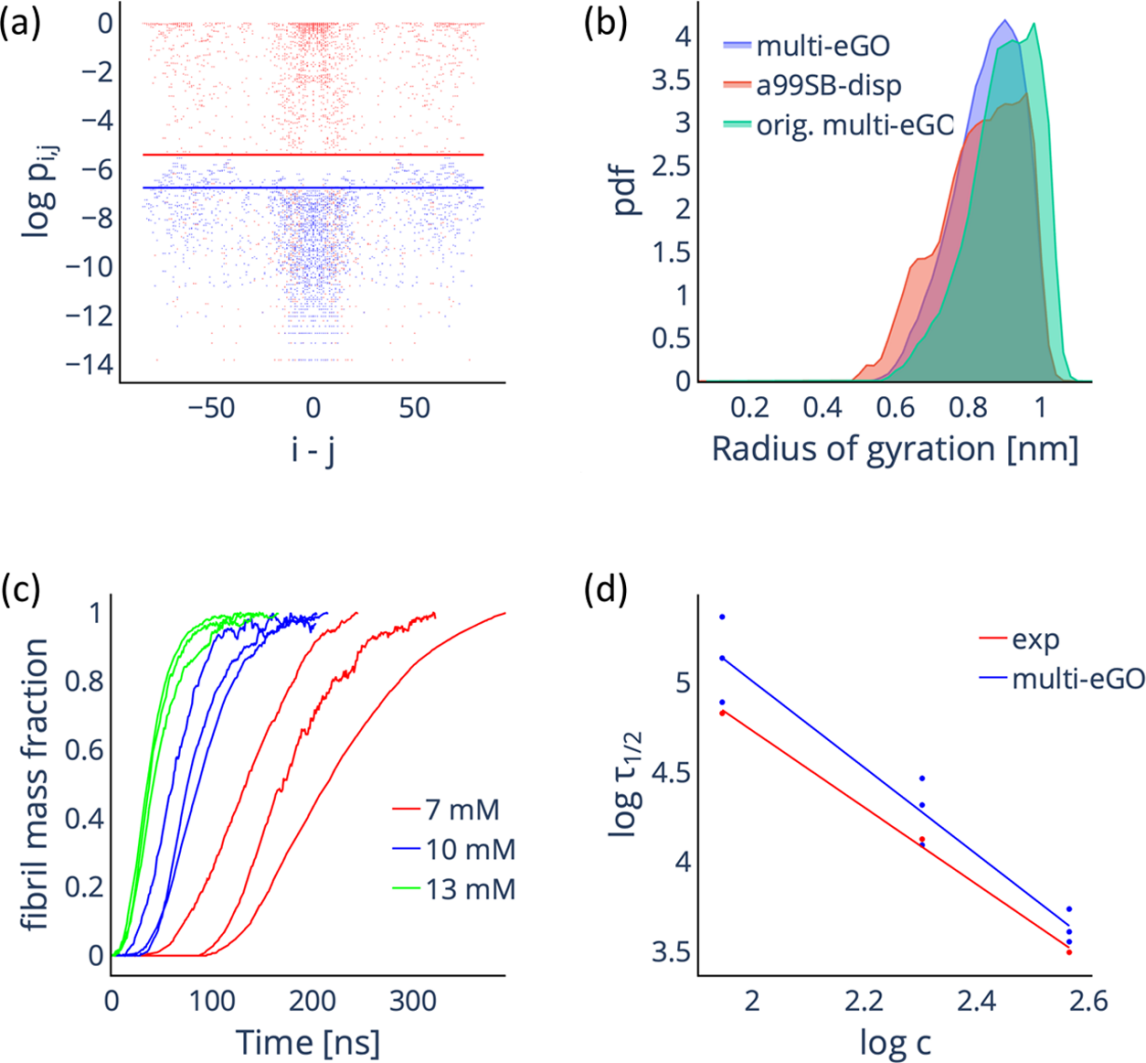
Multi-*e*GO simulations of TTR_105–115_ aggregation as a function of the initial monomer concentration. (a) Comparison of the intermolecular contact probabilities of atom pairs for the training (red dots) and 13 mM RC (blue dots) simulations. The lines represent the *P*_threshold_^MD^ (red) and *P*_threshold_^RC^ (blue) values. (b) Comparison of the radius of the gyration probability density function (calculated using the backbone atoms) for the training monomer (red), reformulated multi-*e*GO (blue), and original multi-*e*GO (green) simulations. (c) Simulated aggregation kinetics. Curves represent the normalized number of monomers involved in an aggregate of at least 10 monomers as a function of nominal simulation time. (d) Log–log plot of the half-times as a function of the initial monomer concentration, experimental values (red) are taken from ref. (18) Both sets can be fitted by a straight line with slopes γ = −2.1 ± 0.1 and γ = −2.4 ± 0.2 for the experimental and simulated data, respectively.

In our previous work,^18^ we showed that multi-*e*GO could simulate the aggregation kinetics of TTR_105–115_ as a function of the initial monomer concentration, qualitatively reproducing the expected kinetics and structural features. After reformulating multi-*e*GO, we replicated these simulations. To train the model, we used the previously generated simulation of the monomer, a simulation of the fibril (with the caveat that having observed the fibril to be unstable in solution, we ran the simulation using positional restraints). We also ran an RC simulation of the monomer and three intermolecular prior simulations (at concentrations of 13, 10, and 7 mM), which are required to weight the intermolecular contacts (cf. Section 2). In Figure 4a, we compare the probabilities for the intermolecular contact pair from the training and the prior simulations. The training simulation showed many highly probable intermolecular contacts due to the stable fibril conformation. On the contrary, the prior simulation displayed low probability contacts resulting from the random collisions.

From the training and RC simulations, we set ε_0_^intra^ to 0.275 kJ/mol by maximizing the agreement between the radius of gyration probability distribution for the monomer training and multi-*e*GO simulations. In Figure 4b, we show the overlap between the radius of gyration probability distributions for the training, original, and reformulated multi-*e*GO simulations. It is apparent how the reformulated multi-*e*GO better reproduces the training simulation. Next, we verified that the same value can be used for ε_0_^inter^, resulting in a stable fibril conformation. Finally, aggregation kinetics were simulated in triplicate with starting monomer concentrations of 13, 10, and 7 mM using 4000 monomers. In Figure 4c, we plotted the fraction of fibril mass as the normalized number of monomers involved in an aggregate of at least 10 monomers as a function of the simulation time. The curves showed the expected sigmoidal shape with an increasing lag time with a decreasing concentration. To compare simulations and experiments, we calculated the log–log of both aggregation half-times and concentrations. Both the experimental and simulation data show a linear trend with comparable slopes of −2.1 ± 0.1 and −2.4 ± 0.2, indicating macroscopically comparable kinetics. As in our previous work, the resulting fibrils lack the central cavity while exhibiting correct antiparallel stacking and head-to-tail lateral growth, allowing us to confirm that the reformulated model can still qualitatively describe the aggregation of the TTR_105–115_ peptide.^18,41^

Simplified models for biomolecular simulations have been developed to overcome the time scale and size limitations of conventional molecular mechanics MD. Structure-based models, often at α-carbon resolution, have been used mainly to study not only protein folding^16^ but also large conformational changes,^47−49^ metamorphic proteins,^50,51^ and the folding upon binding of disordered proteins with different partners.^52,53^ With multi-*e*GO, we aim to develop a platform that, building on the increasing availability of high-quality MD simulations (see, e.g., ref (54)), can then be used to study processes involving multiple molecules and long time scales while maintaining atomistic resolution and, indirectly, some chemical specificity. This work, by introducing a well-defined theoretical framework for learning from both homogeneous and heterogeneous conformational ensembles, is our second step in this direction.

## Notes

Simulations data are publicly available via Zenodo with record 10.5281/zenodo.10033341. The multi-*e*GO code and parameters are publicly available on GitHub at https://github.com/multi-ego/multi-eGO. The beta1 tag is used for a snapshot of the repository on the date of paper submission.

## Abbreviations

MD: molecular dynamics
LJ: Lennard–Jones
RC: random coil
Aβ42: amyloid β42 peptide
GB1: B1 immunoglobulin-binding domain of streptococcal protein G
TTR: transthyretin
PDB: Protein Data Bank
RMSD: root-mean-square deviation
pdf: probability density function
cdf: cumulative distribution function
